# Recombinant RNA-Dependent RNA Polymerase Complex of Ebola Virus

**DOI:** 10.1038/s41598-018-22328-3

**Published:** 2018-03-05

**Authors:** Egor P. Tchesnokov, Parisa Raeisimakiani, Marianne Ngure, David Marchant, Matthias Götte

**Affiliations:** 1 Department of Medical Microbiology and Immunology, University of Alberta, Edmonton, Alberta Canada; 2 Li Ka Shing Institute of Virology at University of Alberta, Edmonton, Alberta Canada

## Abstract

Here we report on the expression, purification and characterization of recombinant ebola virus RNA-dependent RNA polymerase (EBOV RdRp). Active protein complexes composed of the large L protein and viral protein VP35 were isolated from insect cells and analyzed using a short primer/template substrate that allowed benchmarking against related enzymes. RNA synthesis by multiprotein complexes of EBOV, influenza B, respiratory syncytial virus (RSV) and monomeric enzymes of hepatitis C and Zika (ZIKV) viruses required a 5′-phosporylated primer. The minimum length of the primer varied between two and three nucleotides in this system. The EBOV enzyme utilizes Mg^2+^ as a co-factor and the D742A substitution provides an active site mutant that likely affects binding of the catalytic metal ions. Selectivity measurements with nucleotide analogues translate our assay into quantitative terms and facilitate drug discovery efforts. The related EBOV and RSV enzymes are not able to efficiently discriminate against ara-cytidine-5′-triphosphate. We demonstrate that this compound acts like a non-obligate chain-terminator.

## Introduction

Negative-sense RNA viruses such as influenza viruses, Measles virus, Mumps virus, respiratory syncytial virus (RSV), and ebola virus (EBOV) are important human pathogens. Unfortunately, effective antiviral treatments are often not available^1–3^. Viral RNA-dependent RNA polymerases (RdRp) are essential for replication of RNA viruses and represent important drug targets. Despite recent progress in the field^4–8^, the expression of active recombinant RdRp enzymes of negative-sense RNA viruses remains challenging. Influenza (Flu) and vesicular stomatitis (VSV) viral RdRp complexes have been successfully crystallized^4,6,7^ and RNA synthesis of these protein complexes, including the RSV complex, can be monitored in biochemical assays^5,8–11^. However, methods for the study of EBOV RdRp and its inhibition have yet to be devised. The nucleotide analogue (GS-5734) has been shown to be a potent inhibitor of EBOV replication in cell culture^12^. GS-5734 also provided post-exposure protection in non-human primates. The authors noted that isolation and expression of EBOV RdRp has been elusive and utilized the homologous RSV RdRp to demonstrate enzyme inhibition with the triphosphate form of this compound. Here we report the expression, purification, and biochemical characterization of an active, recombinant EBOV RdRp complex. Multiprotein complexes derived from negative-sense RNA viruses RSV^5^ and influenza B (FluB)^6,7^, and monomeric polymerases derived from positive-sense RNA viruses hepatitis C virus (HCV) and Zika virus (ZIKV) served as benchmarks^13,14^.

## Results

### Expression of EBOV RdRp

**Figure 1:**
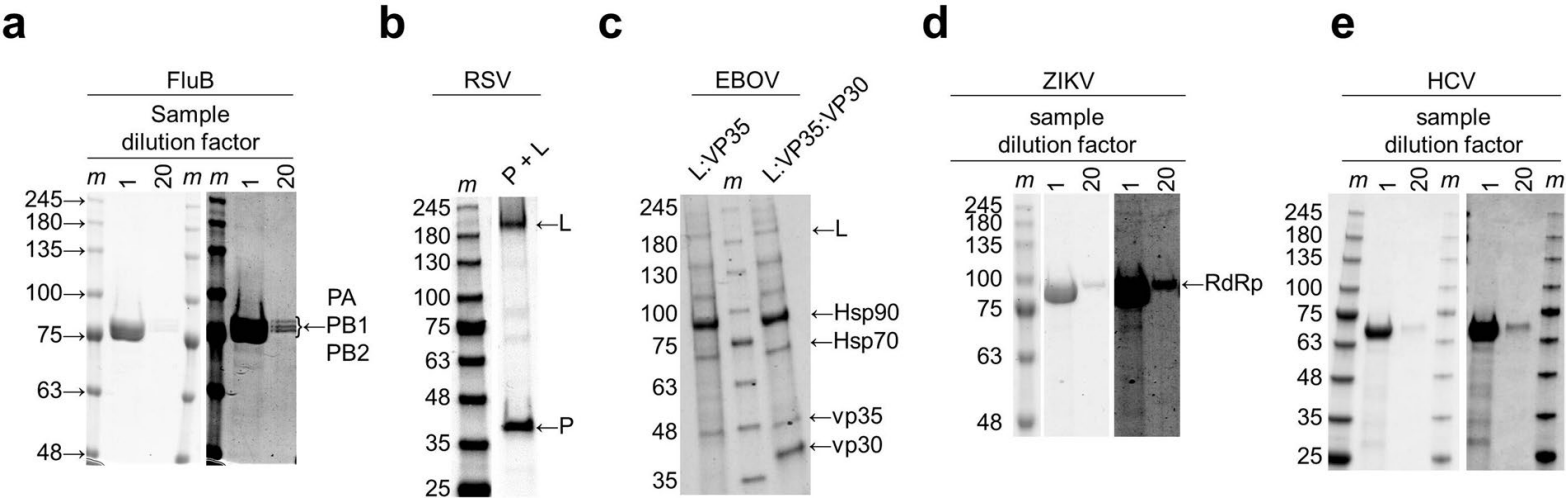
SDS PAGE migration patterns of purified viral proteins. Gels were stained with Coomassie Brilliant Blue G-250 dye. Arrows point to the bands containing the relevant full-length proteins identified by mass spectrometry. “m” represents the marker. (**a**) ~5 μg of FluB protein (*lane* 1) and a 20-fold dilution of the same sample (*lane* 20). The diluted sample shows the three components PA, PB1, and PB2 of the trimeric protein complex. The contrast in the right-hand side sub-panel was uniformly increased to illustrate the purity of the sample. (**b**) The RSV RdRp preparation shows the L and P proteins of the dimeric complex (*lane* P +L). (**c**) The EBOV RdRp preparation shows viral proteins L and VP35 (lane L:VP35), and L,VP35, and VP30 (lane L:VP35:VP30). Cellular proteins Hsp70 and Hsp90 have also been identified. (**d**) and (**e**) ~3–4 μg of HCV and ZIKV proteins, respectively, were loaded on the gel and analysed as in (**a**). The contrast in the right-hand side sub-panels was uniformly increased to illustrate the purity of the samples.

We designed an expression vector that is based on constructs successfully used to produce the trimeric Influenza RdRp complex in insect cells^6,7^. The three components of this complex (PA, PB1, and PB2) are expressed from the same promoter to yield a single polyprotein, which is cleaved by the co-expressed tobacco etch virus (TEV) protease at engineered cleavage sites. The identity of purified proteins was confirmed by mass spectrometry analysis (MS). Purification of the trimeric complex is enabled through affinity chromatography (Fig. 1a, Supplementary Table 1). We used the same approach to produce the dimeric P:L complex of RSV (Fig. 1b), while others express the two proteins from different promoters^5,8^. The requirements for the expression of an active EBOV RdRp complex are unknown. While the L protein is essential for RNA synthesis, the nucleoprotein NP and viral proteins VP30 and VP35 have been considered as possible additional factors^15^. NP is primarily involved in RNA binding, likely independent of the L protein. While the possible contribution of VP30 remains to be defined, VP35 is considered as a functional equivalent of the RSV-associated P protein^15^. Hence, we designed vectors that express the two components L and VP35, or the three components L, VP30, and VP35 from a single promoter. Protein expression in insect cells employed here proved to be the system of choice for the production of both monomeric RdRp from positive-sense RNA viruses and multimeric RdRp from negative-sense RNA viruses. The purity of the FluB RdRp complex is comparable to the previously described method (Fig. 1a)^6,7^. The adaption of this method to produce RSV L:P RdRp complex resulted in a considerably pure protein preparation with only small amounts of heat shock proteins HSP70 and HSP90 from insect cells (Fig. 1b). To remove smaller proteins that are not bound to the complex, the RSV and EBOV protein preparations were concentrated with 100 kDa molecular weight cut-off membranes prior to PAGE analysis. However, EBOV RdRp complexes co-purified with higher amounts of Hsp70 and Hsp90, as well as other insect cell proteins, despite the presence of detergent and 0.5 M NaCl (Fig. 1c, Supplementary Table 1). We therefore refer to these protein preparations as *partially purified*. Co-purification of insect cell proteins despite the stringent purification conditions employed here (extensive column washes, high salt buffer, presence of detergent and the usage of high molecular weight cut-off membranes during concentration step (Supplementary Table 1) suggested that heat-shock proteins may be necessary for stable EBOV RdRp protein preparations. Co-purification with heat shock proteins has been also reported previously by others for the protein preparations of RSV L:P complexes^5^. In fact, Hsp90 is implicated in L protein stability and RdRp complex formation^16–18^. Nevertheless, the presence of insect cell proteins precluded an accurate estimation of the protein complex concentration in the partially purified EBOV RdRp samples. Figure 1c shows that vp30 is absent in preparations with constructs that lack this gene, which provides an additional control. Despite the predicted structural similarity between RSV and EBOV RdRp^4,12^, the dimeric L:VP35 and trimeric L;VP35:VP30 EBOV RdRp complexes express at considerably lower levels then RSV P:L complex. Expression of monomeric RdRp of ZIKV and HCV is shown in Fig. 1d, and 1e, respectively.

### Assessment and optimization of RNA synthesis activity

**Figure 2:**
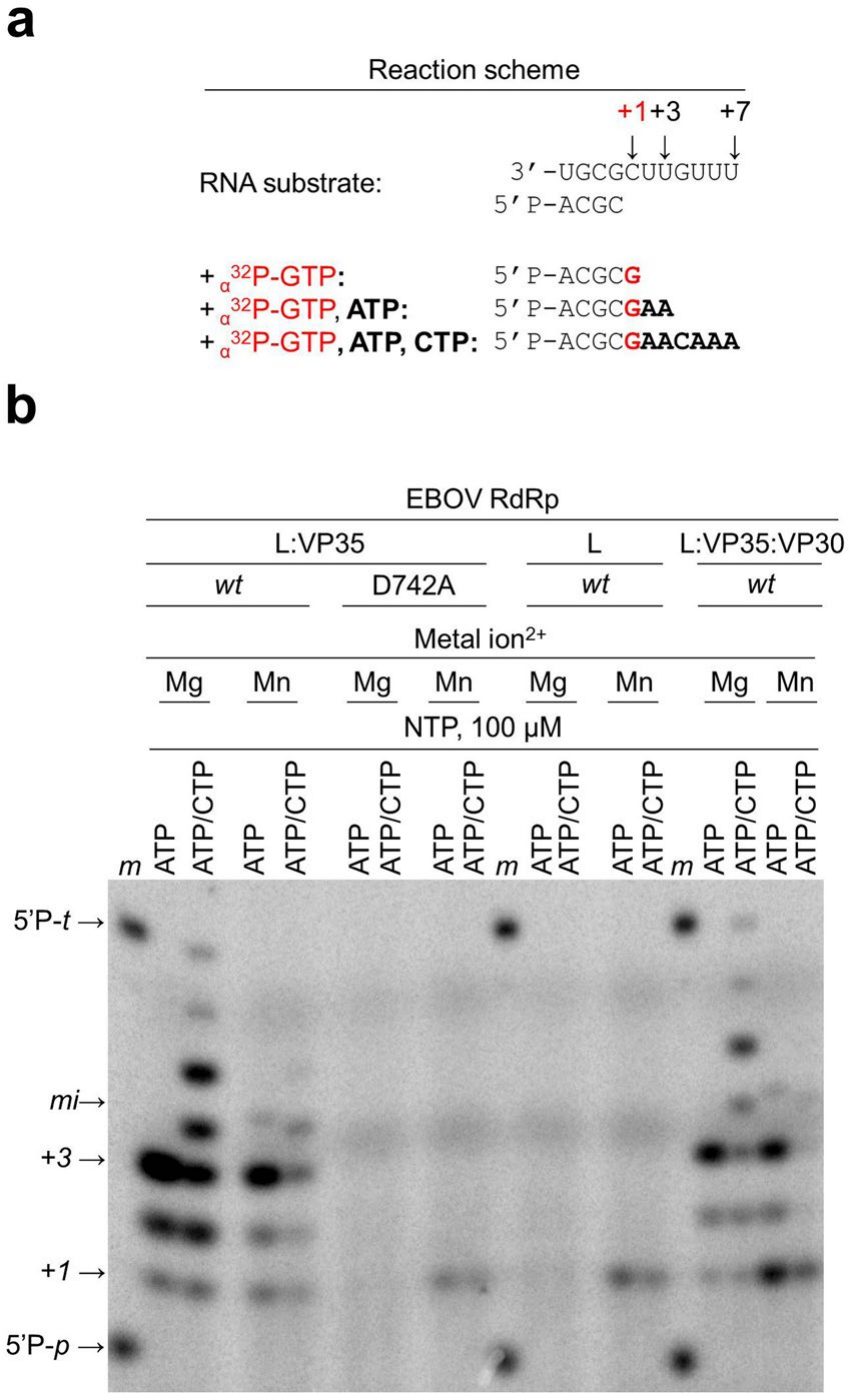
Limited RNA synthesis by EBOV RdRp. 15% denaturing PAGE shows the 5′P-RNA primer and its extension products. (**a**) The reaction scheme shows the nucleotide mixtures that yield one, three or seven nucleotide extensions. (**b**) RNA synthesis in the presence of Mn^2+^ or Mg^2+^ with the binary complex L:VP35 (wt, wild type and the active site mutant D742A), the monomeric L protein and the ternary complex (L:VP35:VP30). *Lane m*, 5′-^32^P-labeled primer (5′P-*p*→) and template (5′P-*t*→) markers. *mi→*, product of misincorporation at position +4.

Based on the related nature of the RSV and EBOV RdRp complexes, we monitored RNA synthesis activity using a tested RSV-derived, RNA model primer/template (P/T) substrate^8^ (Fig. 2). The primer is phosphorylated at its 5′-end and contains four nucleotides that are complementary to the 3′-end of a 11-mer template (Fig. 2a). The template permits incorporation of a radio-labelled nucleotide at position +1, and, depending on the available NTPs, formation of an intermediate product at position +3, and a full-length product at position +7. Activity was tested with the potential catalytic, divalent metal ions Mg^2+^ and Mn^2+^, respectively. In the presence of Mg^2+^, the dimeric complex L:VP35 shows the expected products at position +1, +3, and +7 (Fig. 2b). We obtained essentially the same data with the trimeric complex L:VP30:VP35, indicating that VP30 is not essentially required for RNA synthesis. Mn^2+^ is less efficient in catalyzing the reaction, and, under these conditions, RNA synthesis is more prone to misincorporations. A faint band corresponding to G:A misincorporation at position +4 was detected only in the presence of Mn^2+^ (Fig. 2b, panel L:VP35, sub-panel *wt*, lanes ATP).

Conserved motifs characterize the environment around the active site of RdRp enzymes^19,20^. Motif C (Supplementary Table 1, sequence alignment) is responsible for coordinating metal ions during catalysis^21^. Amino acid substitutions within the Motif C generally result in an inactive enzyme^5,22–24^. In particular, changing the first aspartate of the GDD/N sequence within Motif C was shown to cause ablation of the nucleic acid synthesis in some RdRp^25,26^. The corresponding residue in EBOV RdRp is D742 (Supplementary Table 1). We demonstrate that the EBOV D742A mutant enzyme lacks the ability to extend the primer in the presence of Mg^2+^ (Fig. 2b). In the presence of Mn^2+^, a faint band is seen at position +1. The same result was obtained with the L protein that was expressed in the absence of VP30 and VP35. These conditions may still allow binding of Mn^2+^ but not Mg^2+^. However, the faint band at position +1 suggests that binding of Mn^2+^ is rather inefficient. The lack of longer reaction products further shows that these conditions do not allow repeated cycles of nucleotide incorporation, which involves the sequence of nucleotide binding, catalysis, release of pyrophosphate, and translocation. Together our data suggest that Mg^2+^ is the biologically relevant co-factor for RNA synthesis by EBOV RdRp.

**Figure 3:**
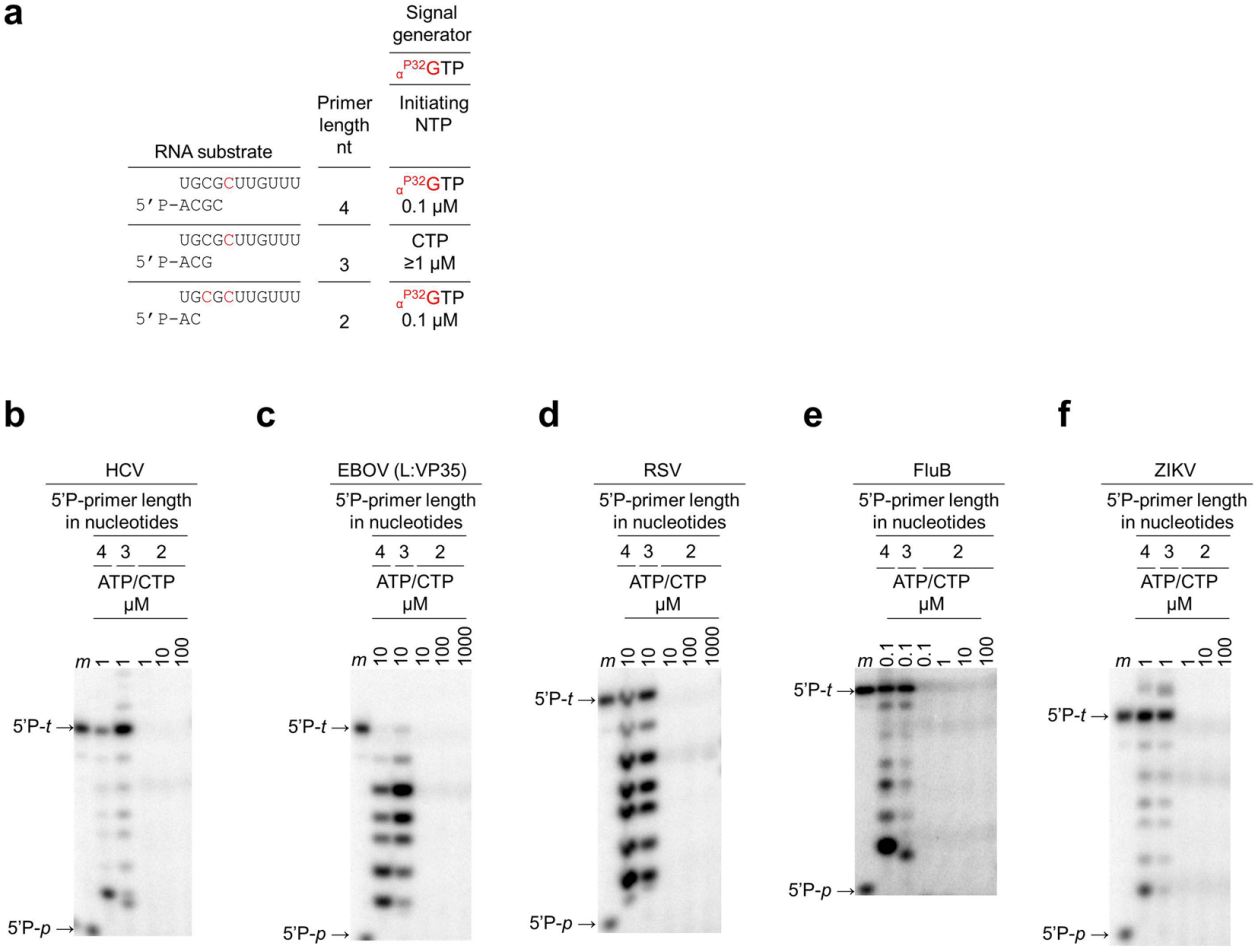
RNA synthesis as a function of primer length. Uncropped images of the panels are shown in Supplementary Fig. 3. (**a**) RNA substrates are shown for different primer lengths: 4, 3, and 2. The reaction conditions are such that the concentration of the first nucleotide to be incorporated at the 3′-end of the primer (*initiating NTP*) changes as a function of primer length (*nts*, nucleotides) used in the reaction mixture. Template positions at which radio-labeled nucleotide is incorporated are illustrated in red. (**b–f**) RNA synthesis by viral RdRp as a function of 5′-phosphorylated primer length. 15% denaturing PAGE migration pattern of 5′-phosphorylated primers of various lengths extended through incorporation of nucleotides. *Lane m*, 5′-^32^P-labeled primer (5′P-*p*→) and template (5′P-*t*→) markers.

For comparative analyses, we optimized assay conditions for RNA synthesis for each of the enzymes used in this study. ZIKV and FluB RdRps showed optimal activity in the presence of 2.5 mM MnCl_2_, while HCV, RSV and EBOV RdRp showed optimal activity at 5 mM MgCl_2_ (Supplementary Figs 1 and 2). 20 mM NaCl was used for all enzyme reactions, which is based on the limitations seen with HCV RdRp. We also considered the length of the primer as an additional reaction parameter and determined the minimum length required for RNA synthesis (Fig. 3). We utilized 5′-phosphorylated primers composed of four, three, and two nucleotides (Fig. 3a). The concentration of the initiating nucleotide differs depending on the length of the primer, which translates in higher yields in reactions containing three-nucleotide primers (Fig. 3b–f).

**Figure 4:**
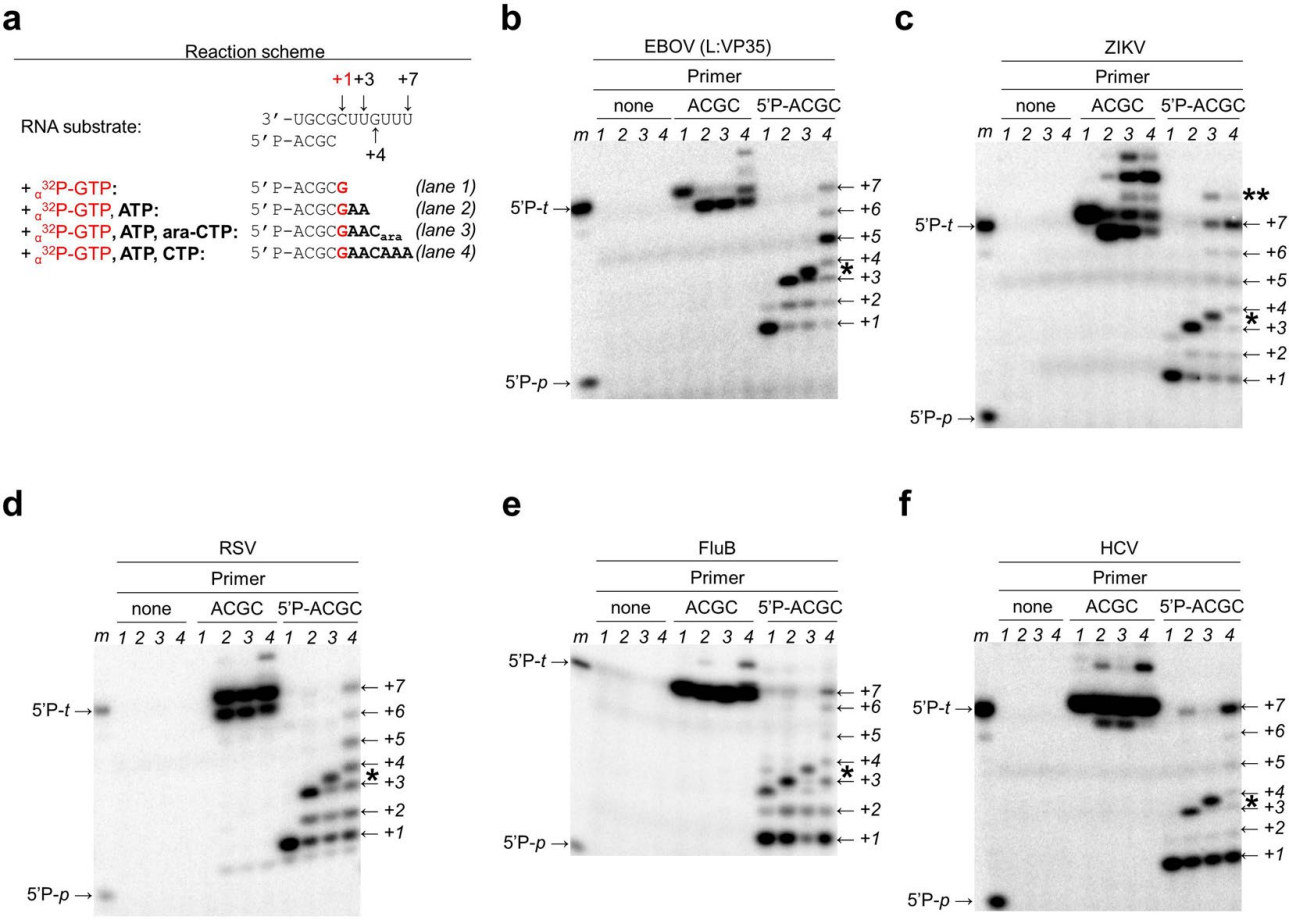
RNA synthesis and inhibition. Due to the differences in relative activities of the enzymes, the contrast in each panel was uniformly adjusted to simplify qualitative comparisons of the patterns across the five enzymes. Uncropped auto-scaled images are shown in Supplementary Fig. 8. (**a**) Reaction scheme for the assay: the primer is extended by one, three, four or seven nucleotides depending on the nucleotide mixture as indicated. (**b–f**) 15% denaturing PAGE migration pattern of products of RNA synthesis in the absence (left panel), in the presence of non-phosphorylated primers (middle panel) and 5′-phosphorylated primers (right panel). *Lane m*, PAGE migration pattern of 5′-^32^P-labeled primer (5′P-*p*→) and template (5′P-*t*→) markers. Asterisk (*) indicates the migration pattern of ara-CMP-terminated primers (*lane 3*). Double asterisk (**) indicates the migration pattern of terminal transferase activity.

Our data show that a tri-nucleotide primer was sufficient for efficient RNA synthesis by each of the aforementioned five viral polymerases, while a di-nucleotide primer did not yield significant amounts of product under these conditions (Fig. 3b–f). This observation is consistent with the proposed transition from a fragile, distributive initiation process to a more stable and processive elongation mode in HCV^27–29^. Our data point to a similar transition in ZIKV, FluB, RSV, and EBOV RdRp (Fig. 3c–f). It is also evident that HCV, ZIKV, and FluB RdRps yield predominantly full-length +7 RNA products. EBOV and to a certain degree also RSV RdRp show several products up to +5 and minor products at +6 and +7, which points to relative deficits in processive RNA elongation. A comparison of lane 1 and 4 (Fig. 4b, panel 5′P-ACGC) reveals that the first product of RNA synthesis (band at “+1” in lane 1) almost completely disappeared when all nucleotides were present in the reaction mixture (+2 to +7, lane 4). Hence, the RNA primer “+1” is utilized by the enzyme as a substrate for nucleotide incorporation at position +2 and so on. The disappearance of the initial product at position +1 illustrates efficient substrate utilization during catalysis. We therefore concluded that the RSV-derived RNA primer template is indeed suitable for testing the RNA synthesis activity by EBOV RNA pol. Most importantly, this primer/template sequence is utilized as a substrate by all five enzymes, which facilitates selectivity measurements.

Depending on the primer length used in our reaction, the initiating nucleotide may also be the radioactively-labelled nucleotide **(**Fig. 3a**)**. RNA synthesis is here limited by the sub-micromolar concentration of _α_^32P^-GTP. Under these conditions HCV RdRp does not initiate RNA synthesis from a di-nucleotide primer (Fig. 3b). Moreover, increasing concentration of nucleotides for subsequent incorporations (positions +2, +3 (ATP) and +4 (CTP)) fails to rescue RNA synthesis. However, RNA synthesis is efficiently initiated with non-labeled nucleotides that can be used at higher concentrations (Supplementary Fig. 3). Under these conditions HCV RdRp initiates RNA synthesis from a dinucleotide primer at 100 μM concentrations of the initiating nucleotide (GTP) (Supplementary Fig. 3b, right panel). FluB shows a very similar pattern (Supplementary Fig. 3e, right panel). None of the remaining enzymes was capable of initiating dinucleotide-primed RNA synthesis to a comparable extent under similar conditions.

### Selectivity of nucleotide incorporation

RNA polymerases are expected to interact with the 2′-OH group of the incoming NTP^28^. Hence, we tested the ability of the EBOV enzyme to accommodate nucleotide analogues 2′β-hydroxy-cytidine-5′-triphosphate (ara-CTP) and 2′-deoxy-cytidine-5′-triphosphate (2′-dCTP) that are modified at this position. The 2′-OH group is in the “up” β-conformation in ara-CTP as opposed to the “down” α-conformation in natural CTP pools, while the 2′-OH group is absent in 2′-dCTP (Supplementary Fig. 4). Incorporation of ara-CTP or 2′-dCTP is enabled at template position + 4 (Fig. 4a). Depending on the nucleotide mixture, products are expected at positions +1, +3, +4, and +7. Reactions were performed without a primer, with a non-phosphorylated primer, and with a 5′-phosphorylated primer as described above (Fig. 4b–f). No radio-labeled products were detected in the absence of a primer. Although we identified labelled reaction products with a non-phosphorylated primer, the nature of this activity remains elusive. Product formation is here rather independent of the template sequence. Similar patterns are seen with each of the five enzymes, suggesting that the lack of a 5′-phosphate group and not the nature of the enzyme causes these effects. In contrast, data obtained with the 5′-phosphorylated primer yielded the expected reaction products (Fig. 4b–f). The product at position +1 is a 5-mer with incorporated _α_^32^P-GTP (lane 1), and the product at position +3 is a 7-mer that shows incorporation of ATP at positions +2 and +3 (lane 2). The reaction with ara-CTP shows a product that migrates between positions +3 and +4 (lane 3). The lack of longer reaction products demonstrates effective chain-termination, which is also the proposed mechanism for the triphosphate form of GS-5734^12^. Longer products, including the full-length product are seen in the control in which ara-CTP was replaced with CTP (lane 4). With regards to the incorporation of ara-CTP, RSV, FluB, and HCV enzymes show the same pattern as the EBOV enzyme (Fig. 4b, d–f). In contrast, ZIKV RdRp incorporates ara-CTP and yields in addition the full-length product at position +7 (full-length) and a product at position +8 that is likely a result of terminal transferase activity (Fig. 4c). These data are indicative of incomplete chain-termination.

The above assay was modified to measure selective incorporation of the natural CTP substrate over ara-CTP and 2′-dCTP, respectively (Supplementary Figs 5–7). The selectivity values follow the order HCV > FluB > ZIKV > EBOV > RSV and HCV > ZIKV > EBOV > RSV > FluB, respectively (Table 1). FluB RdRp shows the lowest selectivity value with respect to 2′-dCTP, while it ranks second with respect to ara-CTP. A ~100-fold spread in 2′d-CTP and ara-CTP selectivity values exhibited by the five viral RdRp enzymes illustrates virus-specific requirements for nucleotide substrates during RNA genome replication.

**Table 1:** Selectivity values for ara-CTP and 2′d-CTP nucleotide analogues.

	Nucleotide substrate										
	CTP			2′dCTP				ara-CTP			
	*V*_max_^a^ p.frac.	*K*_m_^a^ μM	*Eff*.^b^	*V*_max_ p.frac.	*K*_m_ μM	*Eff*.	***Sel***.^c^	*V*_max_ p.frac.	*K*_m_ μM	*Eff*.	***Sel***.
**HCV**											
	n = 4			n = 3				n = 3			
	0.97	0.00060	1617	0.84	0.34	2.5	**654**	0.95	0.059	16	**100**
± ^d^	0.0083	0.000034		0.014	0.032			0.01	0.0040		
%err.^e^	1	6		2	9			1	7		
**ZIKV**											
	n = 3			n = 3				n = 3			
	0.96	0.0032	300	0.83	0.25	3	**90**	0.95	0.026	37	**8**
±	0.0064	0.00012		0.012	0.021			0.011	0.0019		
%err.	1	4		1	8			1	7		
**EBOV**											
	n = 3			n = 3				n = 3			
	0.82	1.2	0.68	0.59	63	0.01	**73**	0.77	3.2	0.24	**3**
±	0.014	0.11		0.022	7.7			0.015	0.27		
%err.	2	9		4	12			2	8		
**RSV**											
	n = 3			n = 3				n = 3			
	0.70	0.049	14	0.27	0.45	0.60	**24**	0.74	0.035	21	**0**.**68**
±	0.0088	0.0042		0.0053	0.048			0.012	0.0038		
%err.	1	9		2	11			2	11		
**FluB**											
	n = 6			n = 7				n = 5			
	0.88	0.0038	232	0.38	0.010	38	**6**	0.77	0.077	10	**23**
±	0.012	0.003		0.0078	0.0015			0.012	0.0061		
%err.	1	8		2	15			2	8		

^a^All reported values have been calculated on the basis of a 12-data point experiment repeated at least 3 times (n = ) for natural substrate and the two substrate analogues for each of the five enzymes).

^b^*Eff*., efficiency of nucleotide substrate incorporation during RNA synthesis; calculated as a ratio of the Michaelis-Menten parameters *V*_max_ to *K*_m_.

^c^*Sel*., selectivity of a viral RdRp for a given nucleotide substrate analogue; calculated as the ratio of the efficiency of CTP incorporation to the efficiency of the respective nucleotide substrate analogue incorporation.

^d^Standard error associated with the fit.

^e^Percent error.

## Discussion

Viral RdRp is viewed as a logical target for the development of novel antiviral drugs to treat infection with RNA viruses. RdRps of positive-sense RNA viruses such as HCV RdRp have been validated in this regard, and recent advances in expression and biochemical characterization of RdRp associated with negative-sense RNA viruses show promise^8,12,30^. Here we employed the baculovirus expression system combined with affinity tag purification to produce partially purified functionally active EBOV RdRp. Related enzyme complexes served as benchmarks. The near homogeneity of both the FluB and RSV RdRp protein complex preparations suggested that an expression system based on a single promoter may be beneficial for the production multiprotein complexes. Although we succeeded to produce active EBOV RdRp complexes using the same approach, the yield is considerably reduced and the samples contained several insect cell proteins including Hsp70 and Hsp90. Hsp90 has been shown to play a role in L protein stability and RdRp complex formation^16,18^. Hence, we focused on the functional characterization of protein preparations as shown in Fig. 1. Our data show that a minimum functional complex is L:VP35. L alone was not active, while L:VP35:VP30 and L:VP35 exhibited essentially the same pattern of RNA synthesis. Based on EBOV-specific mini-genome studies it was concluded that EBOV ribonucleoprotein (RNP) complex contains NP, VP30, VP35 and L with L and VP35 constituting a functional polymerase complex^15^. VP35 was proposed to be a functional equivalent of P proteins of related viruses such as RSV and VSV. In fact, the P protein was shown to enhance RNA synthesis by VSV and is an essential component of the RSV RdRp L:P complex, which is in line with our observations^5,11^.

We further demonstrated that RdRp complexes from EBOV, RSV, and FluB, as well as the monomeric HCV and ZIKV enzymes accepted the same model substrate that was initially described in the context of studies with RSV RdRp L:P complex^8^. We observed a favourable comparison of the *K*_m_ values for CTP obtained here for RSV RdRp (0.049 ± 0.004 μM) and the corresponding value obtained by Deval *et al*.^8^ (0.057 ± 0.009 μM). In addition, the selectivity value for ara-CTP in the context of HCV RdRp (100 fold) compares with the previously reported value (140 fold)^31^. Therefore, this method represents a stable reference system and provides a tool to compare biochemical properties of different RdRp enzymes. EBOV and RSV polymerase complexes show a preference for Mg^2+^ ions as catalytic cofactors, FluB and ZIKV enzymes utilize Mn^2+^, and HCV RdRp can utilize either Mg^2+^ or Mn^2+^.

All five enzymes initiate RNA synthesis from a minimum of a 3-mer RNA primer. HCV RdRp was able to utilize a di-nucleotide primer only with high concentration of the initiating nucleotide, which is well-documented in the literature^27,31^. Unexpectedly, specific base-dependent RNA synthesis patterns could be observed only with a 5′-phosphorylated primer. For each of the five RdRp employed in this study, the use of 5′-non-phosphorylated primers yielded reaction products that migrated much higher than expected. While the underlying mechanism remains elusive, this activity is largely independent of the available nucleotides in the reaction mixture. The use of a 5′-phosphorylated primer has been reported in only a few cases, including studies that involved RSV RdRp and mitochondrial RNA polymerase^8,32,33^. Our data show that short model substrates require a 5′-phosphorylated primer to form a biologically relevant elongation complex, which is important for the development of plate-based high-throughput assays that do not reveal the nature of the reaction products.

Active elongation complexes were also analyzed with respect to nucleotide selectivity. To maintain RNA genome fidelity, viral RdRp must select ribonucleotide triphosphates (NTP) against deoxyribonucleotide triphosphates (dNTP) during RNA synthesis. In fact, the five RdRps are all capable of incorporating dNTPs; albeit with varied efficiencies. FluB RdRp exhibited the lowest dNTP selectivity (6-fold, or 100 times lower than HCV RdRp). While this level of selectivity appears to be very poor, it is likely balanced by much higher intracellular NTP pools when compared to dNTP^34^. Regardless, the biological relevance of our virus specific observations remains to be investigated; however, it has been reported that RNA viruses are able to incorporate dNTP in their genome. The Dengue virus genome contained on average ~7 DNA residues per genome^35^. Ara-CTP is the triphosphate form of the approved anti-cancer drug cytarabine. Its selectivity value for HCV RdRp reported here (100) correlates well with the previously reported value (140)^31^. Similar to our measurements with 2′d-CTP, selectivity for ara-CTP also varied among the five RdRp analysed and the order is different. The related EBOV and RSV enzymes show poor discrimination against ara-CTP, which promotes incorporation of the inhibitor under biologically relevant conditions in the presence of competing CTP pools. Moreover, once incorporated, the inhibitor acts like a non-obligate chain-terminator. The ability to cause chain-termination depends on the interaction between the nucleotide analogue and the specific enzyme. Although ZIKV RdRp is able to incorporate ara-CTP, RNA synthesis is in this case only partially inhibited.

In conclusion, the present work provides novel biochemical tools for the study of efficiency and fidelity of RNA synthesis by EBOV RdRp. Comparative studies with related enzymes can potentially deliver a platform for drug discovery and development efforts at a larger scale. The use of the same primer/template system facilitates the discovery of both selective inhibitors and other compounds with a broader spectrum of activities. Moreover, the expression of active EBOV RdRp enables mechanistic studies. Experiments with the homologous RSV RdRp suggest that GS-5734 may act as a chain-terminator^12^. The assays described in this study will help to validate the triphosphate form of GS-5734 as a bona fide inhibitor of EBOV RdRp.

## Methods

### Protein expression, purification and RNA synthesis assays

All virus and viral protein specific information as well as pertinent details of protein expression, purification and RNA synthesis assays are summarized in Supplementary Table 1. HCV RdRp was produced in *Escherichia coli* as previously described^36^. The pFastBac-1 (Invitrogen) plasmid with the codon-optimized synthetic DNA sequences coding for viral proteins or protein complexes (GenScript) was used as a starting material for viral protein expression in insect cells (Sf9, Invitrogen). The construct for the production of the active site mutant D742A EBOV RdRp (L:VP35) was generated with a mutagenic primer. The codon for the amino-acid substitution was introduced with Phusion High-Fidelity DNA polymerase (ThermoScientific) according to the manufacturer’s recommendations. The sequence was confirmed at Molecular Biology Facility at University of Alberta, Canada. We employed the MultiBac (Geneva Biotech) system according to protocols provided by Drs. Garzoni, Bieniossek and Berger^37,38^. Viral proteins and protein complexes were purified using the His- or Strep (Supplementary Table 1) tag-affinity chromatography according to the manufacturer’s specifications (IBA and ThermoScientific, respectively). The overall yield of functional partially purified EBOV RdRp (100–150 μL per 3 L of insect cell culture) precluded additional purification steps. Instead, we applied stringent purification conditions, which included extensive column washes (60 column volumes) in the presence of high salt and the use of 100 kDa molecular weight cut-off membranes to concentrate the protein preparation (Supplementary Table 1). The identity of the purified viral proteins and protein complexes was confirmed by mass spectrometry (MS) analysis (Dr. Jack Moore, Alberta Proteomics and Mass Spectrometry). Briefly, portions of the SDS-PAGE gel were submitted for in-gel protein identification^39^. The excised gel bands were digested with trypsin after reduction and alkylation. Tryptic peptides were subsequently analyzed by LC (liquid chromatography)-MS/MS using an LTQ Orbitrap XL hybrid mass spectrometer (Thermo Scientific). Data processing and analysis was done using Proteome Discoverer 1.4 (Thermo Scientfifc) and Sequest (Thermo Scientific), respectively.

The presence of insect cell proteins precluded reasonable estimation of the protein complex concentration in the partially purified EBOV RdRp samples. The RdRp complexes of FluB and RSV did not show significant presence of insect cell proteins (Fig. 1). Therefore, their protein concentration as well as the concentration of ZIKV and HCV RdRp was determined based on calculated extinction coefficients (GPMAW-lite free software, Alphalyse). Amicon-Ultra (Millipore) centrifugal membranes were used to concentrate purified protein samples.

### Data acquisition, quantification and analysis

The reactions conditions were chosen so that the RNA synthesis product formation was linear with respect to time. Thus, a 30-minute time point was chosen to stop all the reactions involving titration of nucleotide substrates. Reaction substrates and products were resolved through denaturing 8 M urea 15 or 20% PAGE, visualized and quantified by phosphorimaging. Incorporated nucleotide product fraction was plotted versus nucleotide substrate concentrations and fitted to Michaelis-Menten equation using Prism software (GrapPad).

### Data Availability Statement

All data generated or analysed during this study are included in the published article (and its Supplementary Information files). Raw numerical values representing PAGE phosphorimager signal quantification are available from the corresponding author on reasonable request.

## Electronic supplementary material

Supplementary Information
