# Supplementary material for: Recombinant RNA-Dependent RNA Polymerase Complex of Ebola Virus
Source: Sci Rep. 2018 Mar 5;8:3970. doi: 10.1038/s41598-018-22328-3 (PMC5838098; doi:10.1038/s41598-018-22328-3)
Supplement: Supplementary file 1 — Supplementary Information [file 41598_2018_22328_MOESM1_ESM.pdf]

Supplementary Information

Recombinant RNA-Dependent RNA Polymerase Complex of Ebola Virus

Egor P. Tchesnokov[1], Parisa Raeisimakiani[1], Marianne Ngure[1], David Marchant[1,2] and Matthias Götte[1,2*]

[1]Department of Medical Microbiology and Immunology, University of Alberta, Edmonton, Alberta, Canada. [2]Li Ka Shing Institute of Virology at University of Alberta, Edmonton, Alberta, Canada. Correspondence and requests for materials should be addressed to M.G. should be addressed to M.G. (email: gotte@ualberta.ca)

Supplementary Table 1. Detailed information regarding viral RdRp production and RNA synthesis assays.

VIRAL INFO						
family	*Flaviviridae*		*Orthomyxoviridae*	*Pneumoviridae*	*Filoviridae*	
genus	*Hepacivirus*	*Flavivirus*	*Influenza B*	*Orthopneumovirus*	*Ebolavirus*	
species	*Hepatitis C virus*	*Zika virus*	*Influenza B virus*	*Human respiratory syncytial virus*	*Zaire ebolavirus*	
virus	Hepatitis C	Zika	Influenza B	human respiratory syncytial virusA2	Ebola	
strain or isolate	AJ238799	ZikaSPH2015: KU321639	B/Memphis/13/03	A2	KR534507.1	
protein	CAB46677.1	ALU33341.1	PA:AAU94844<br>PB1:AAU94857<br>PB2:AAU94870	P:AAB59853<br>L: AAA84898	VP35: AKG65095<br>VP30:AKG65100<br>L:AKG65102	
EXPRESSION CONSTRUCT, PROTEIN PRODUCTION						
coding region	CAB46677.1<br>2420-3010	synthetic DNA codon-optimized for expression in insect cells (GenScript)				
design	Ferrari *et al.*, 1999	adapted from Zhao *et al.*, 2015	based on Reich *et al.*, 2014	adapted from Reich *et al.*, 2014		
design details	Polyprotein:<br>2420-2989<br>[SMS-RPR]	Polyprotein:<br>2525-3416<br>[GETL-LGEE]				
vector	pET21b	pFastBac1, Bacmid				
composition	NS5b<br>Δ21<br>C-terminus	NS5 (domains):<br>Methyltransferase<br>linker<br>RdRp	PA<br>PB1<br>PB2	P<br>L	vp35<br>vp30<br>L	vp35<br>L
host	*E. coli*	*Spodoptera frugiperda (Sf9)*				
sequence alignment	Structure-based alignment of viral RdRp (Chimera, UCSF[1]), *top panel*. Sequence-based alignment of vesicular stomatitis, RSV and Ebola L proteins (T-coffee[2] - http://tcoffee.vital-it.ch/apps/tcoffee/do:regular coffee), *bottom panel*. The alignments were rendered using ESPript[3] - http://espript.ibcp.fr. Numbers above the alignment refer to the HCV residues that are in contact with the 2'-OH of the incoming nucleotide (pdb:4WTA[4]). Number below the alignment (742) refers to the Ebola Lprotein residue that was mutated to alanine.<br>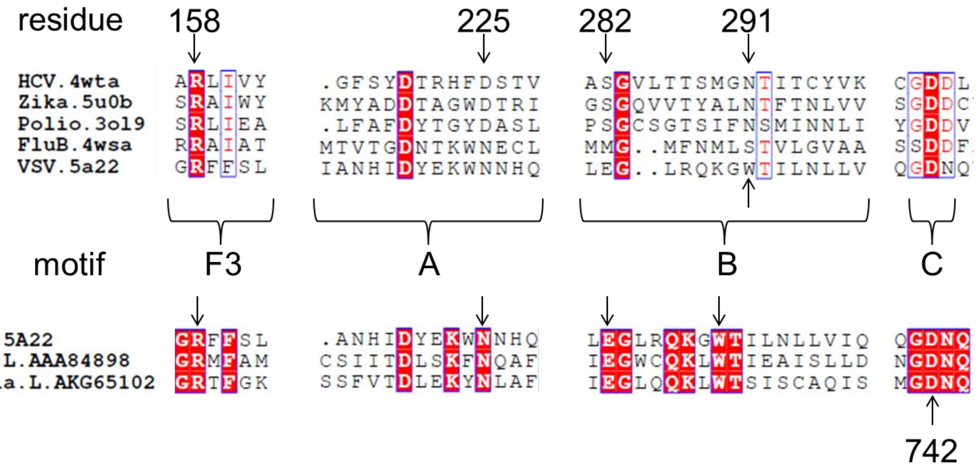					

PURIFICATION						
AFFINITY TAG	Histidine	Streptavidin	Streptavidin	Histidine		
terminus	C	N	C	N		
location	NS5b	NS5	PB2	P	vp35	vp35
WASH BUFFER	NaCl, 500 mM<br>40 cv	NaCl, 1000 mM<br>60 cv	NaCl, 1000 mM<br>60 cv	NaCl, 500 mM<br>60 cv		
Tween 20				0.01, %		
Concentrator	30 kDa	50 kDa	30 kDa	100 kDa		
STORAGE BUFFER	Tris, 50 mM, pH 8, NaCl, 75 mM, TCEP, 2 mM, Glycerol, 50%, final					
RNA SYNTHESIS REACTION MIXTURE						
buffer	Tris, 25 mM, pH 8, NaCl, 20 mM, TCEP, 2 mM					
RNA substrate	Template, 1 μM, Primer, 200 μM					
NTP substrate	1 μM	1 μM	0.1 or 1 μM	10 μM	100 μM	
enzyme	0.4 μM	0.1 μM	0.1 μM	0.2 μM	2 uL	2 uL
reaction start	$MgCl_2$,<br>5 mM	$MnCl_2$,<br>2.5 mM	$MnCl_2$,<br>2.5 mM	$MgCl_2$,<br>5 mM		
stop	Formamide, 95%, EDTA, 25 mM					

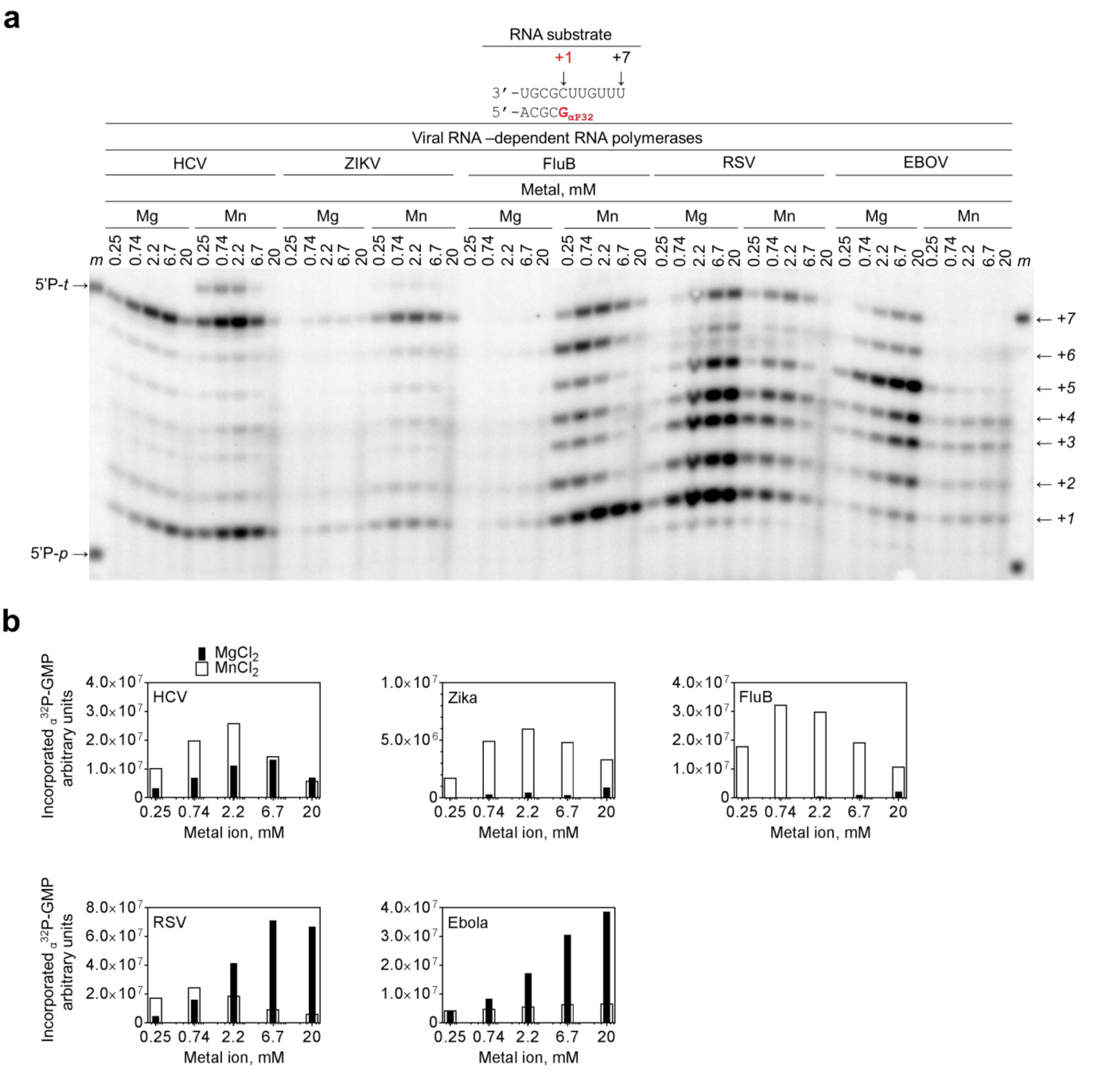

Supplementary Figure 1. RNA synthesis by viral RdRp as a function of di-valent metal ions concentration. (**a**) 15% denaturing PAGE migration pattern of 5’P-RNA primers extended through incorporation of nucleotides. RNA substrate consists of a 5’-phosphorylated primer and 11-nucleotide template. The template permits incorporation of a radio-labelled nucleotide ($_{\alpha}{}^{32}$P-GTP, **G$_{\alpha P32}$**), which effectively labels the primer-extended products of the RNA synthesis. The reaction mixture contains ATP and CTP (**Supplementary Table 1**) nucleotide substrates which allows synthesis of a fully extended primer product position +7. *Lane m*, PAGE migration pattern of 5’-^{32}P-labeled primer (5’P-*p*→) and template (5’P-*t*→) markers. (**b**) Graphical representation of the extent of RNA synthesis by viral RdRp as a function of di-valent metal ions concentration.

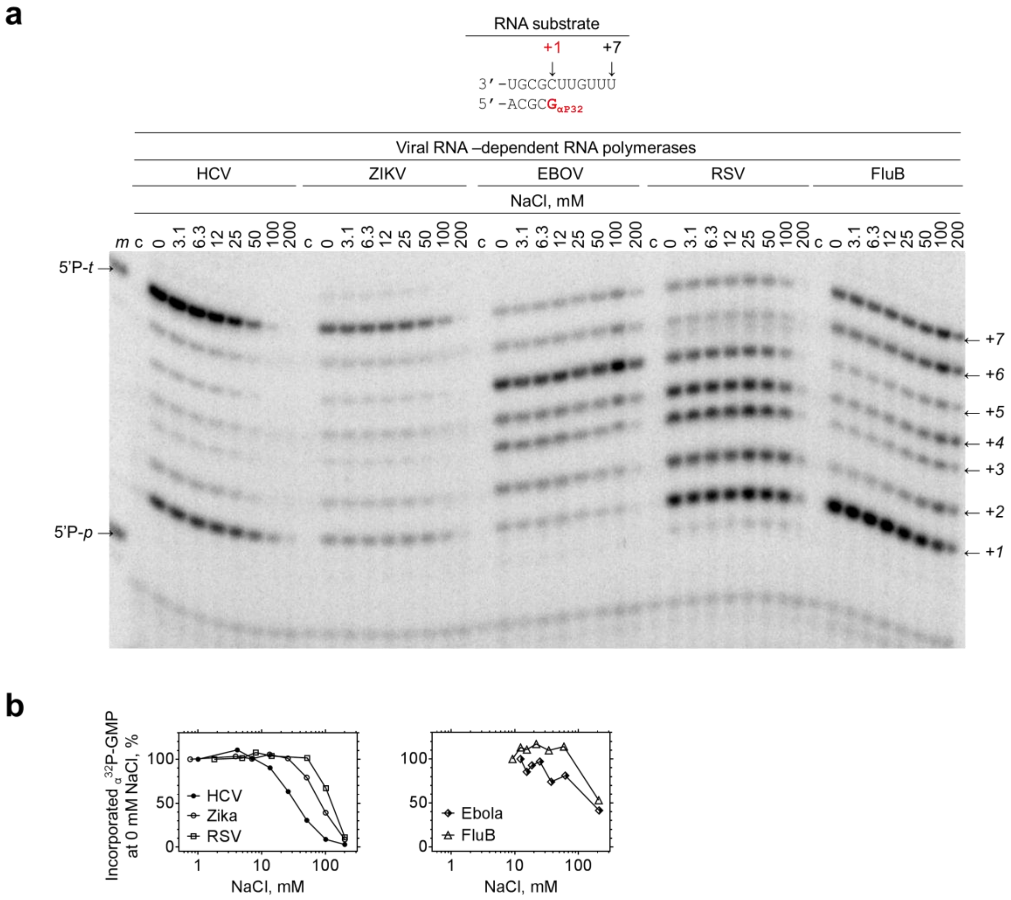

Supplementary Figure 2. RNA synthesis by viral RdRp as a function of mono-valent metal ion concentration. (**a**) 15% denaturing PAGE migration pattern of 5’P-RNA primers extended through incorporation of nucleotides. RNA substrate consists of a 5’-phosphorylated primer and 11-nucleotide template. The template permits incorporation of a radio-labelled nucleotide ($_{\alpha}^{32}$P-GTP, $G_{\alpha P32}$), which effectively labels the primer-extended products of the RNA synthesis. The reaction mixture contains ATP and CTP (**Supplementary Table 1**) nucleotide substrates which allow synthesis of a fully extended primer product position +7.
Lane m, PAGE migration pattern of 5’-^{32}P-labeled primer (5’P-*p*→) and template (5’P-*t*→) markers. *Lane c*, RNA synthesis by viral RdRp in the absence of di-valent metal ions. (**b**) Graphical representation of the extent of RNA synthesis by viral RdRp as a function of mono-valent metal ion concentration.

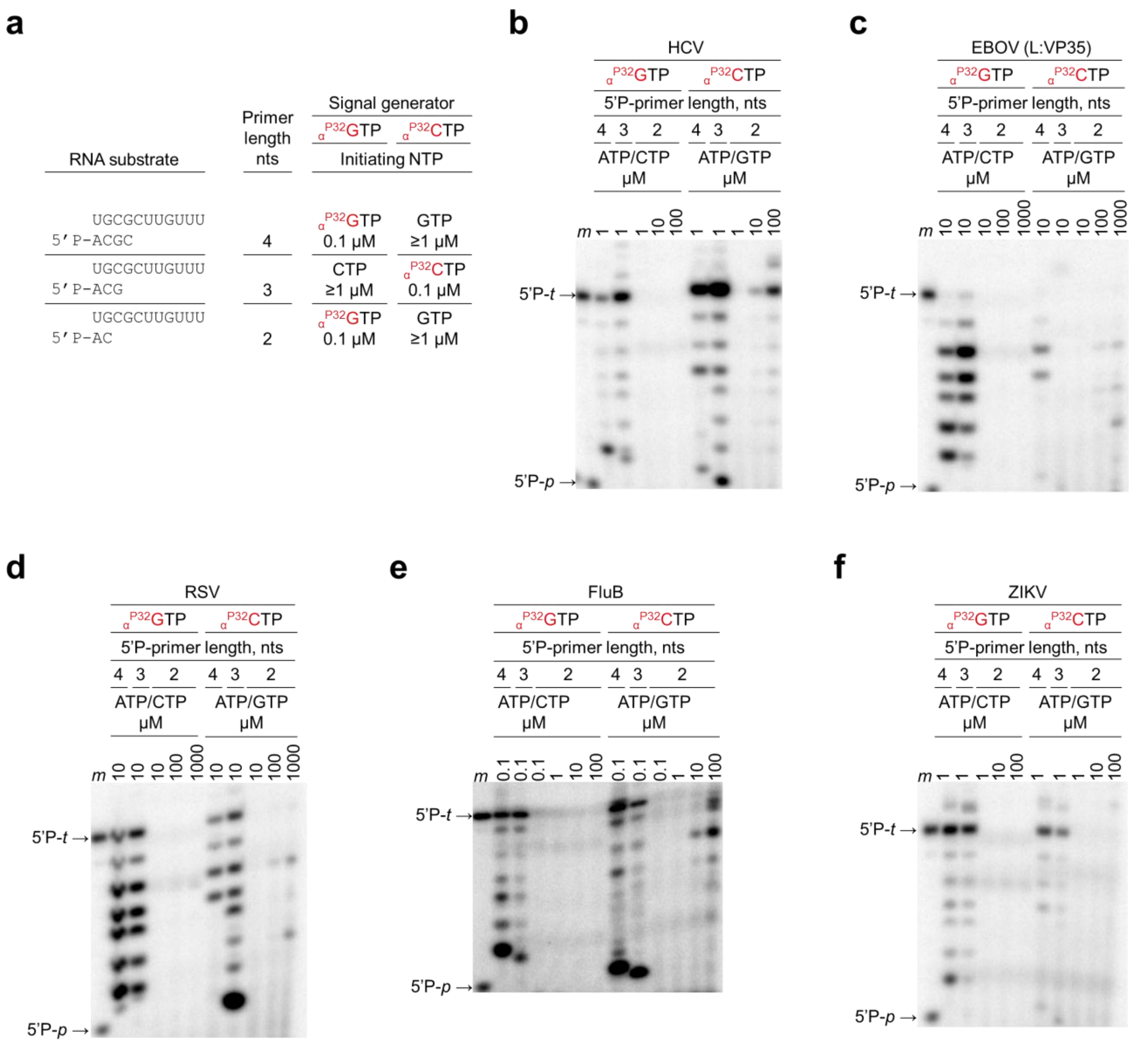

Supplementary Figure 3. RNA synthesis as a function of primer length. (**a**) RNA substrates are shown for different primer lengths: 4, 3, and 2.The reaction conditions are such that the concentration of the first nucleotide to be incorporated at the 3'-end of the primer (*initiating NTP*) changes as a function of primer length (*nts*, nucleotides) used in the reaction mixture. (**b-f**) RNA synthesis by viral RdRp as a function of 5'-phosphorylated primer length. 15% denaturing PAGE migration pattern of 5'-phosphorylated primers of various lengths extended through incorporation of nucleotides including radio-labeled ${}_{\alpha}{}^{32P}$-GTP (left panel) or ${}_{\alpha}{}^{32P}$-CTP (right panel). *Lane m,* 5'-^{32}P-labeled primer (5'P-*p*→) and template (5'P-*t*→) markers.

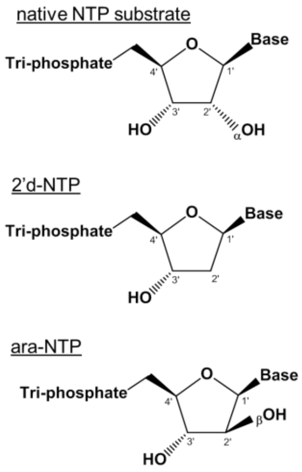

Supplementary Figure 4. Chemical structures of cytidine nucleotide substrate analogues used in the study.

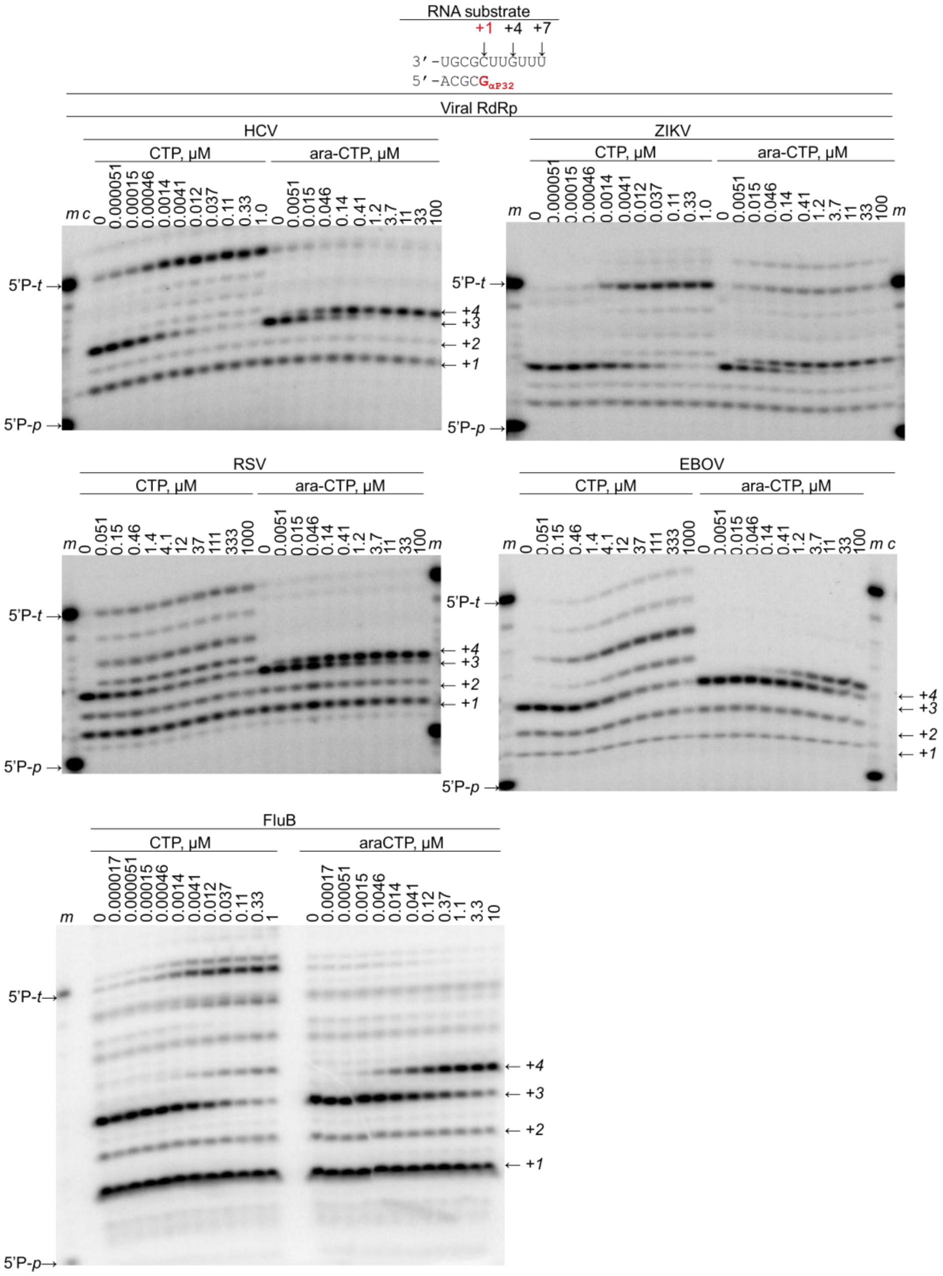

Supplementary Figure 5. RNA synthesis by viral RdRp as a function of incorporation of ara-CMP at position +4 with respect to RNA template. 20% denaturing PAGE migration pattern of products of reactions containing titrated nucleotide substrate concentrations of either CTP or ara-CTP. *Lane m*, PAGE migration pattern of 5'-^{32}P-labeled primer (5'P-*p*→) and template (5'P-*t*→) markers.

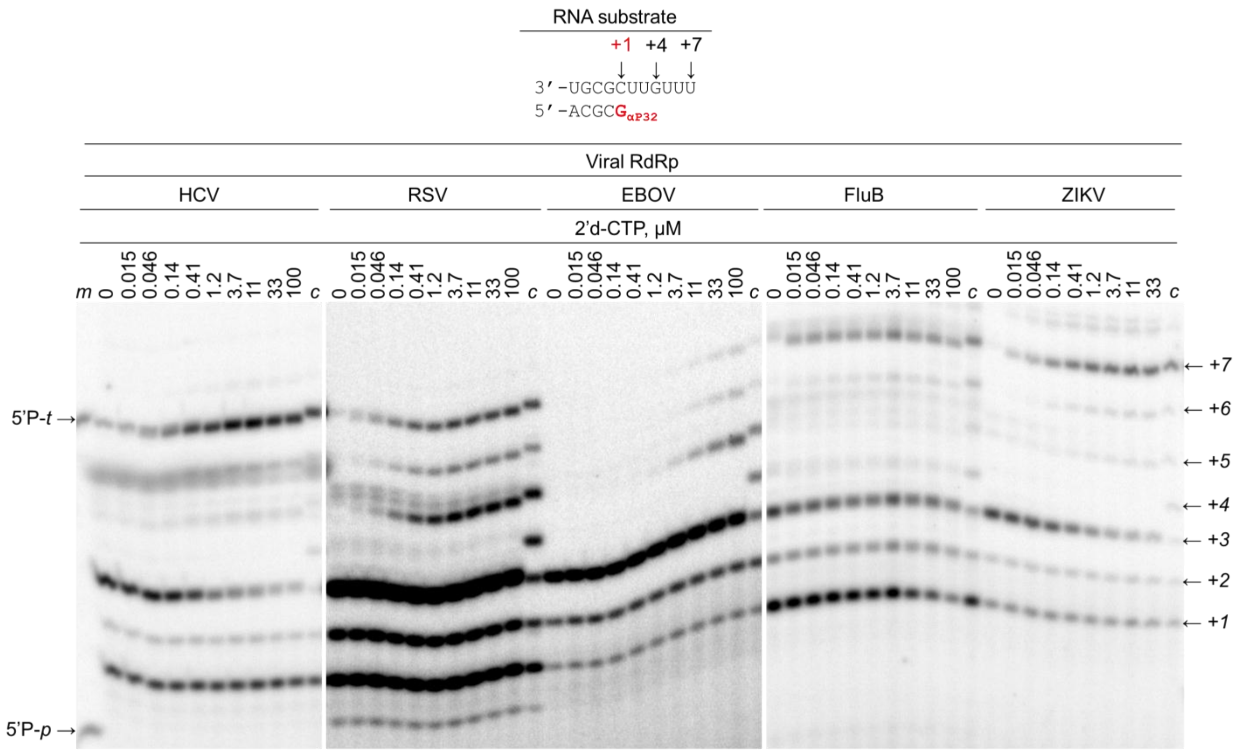

Supplementary Figure 6. RNA synthesis by viral RdRp as a function of incorporation of 2'd-CMP at position +4 with respect to RNA template. 20% denaturing PAGE migration pattern of products of reactions containing titrated nucleotide substrate concentrations of either CTP or ara-CTP. *Lane m*, PAGE migration pattern of 5'-^{32}P-labeled primer (5'P-*p*→) and template (5'P-*t*→) markers.

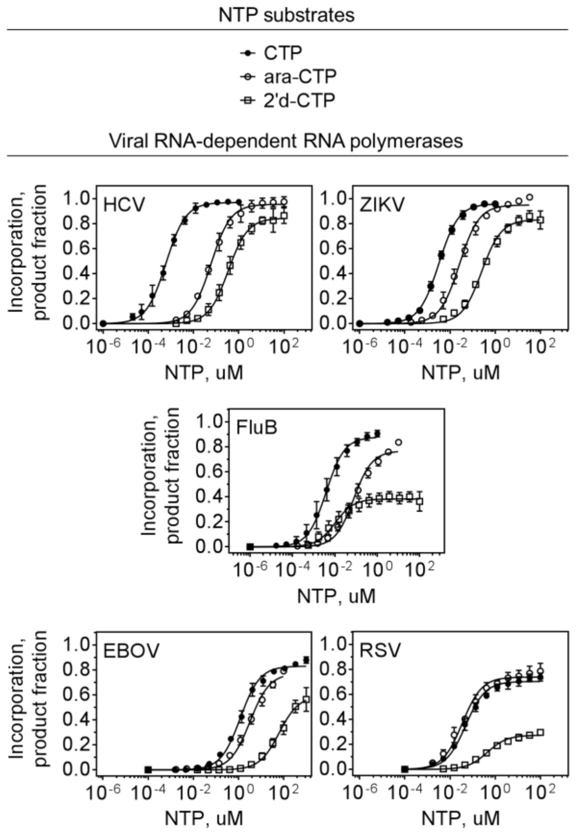

Supplementary Figure 7. Data quality and analysis of incorporation of ara-CMP and 2’d-CMP during RNA synthesis by viral RdRp. Error bars represent standard deviation of the data points determined on the basis of at least three independent experiments. Data points were fit to a Michaelis-Menten equation using Prism software (GrapPad). Michaelis-Menten parameters calculated from the fits are reported in **Table 1**.

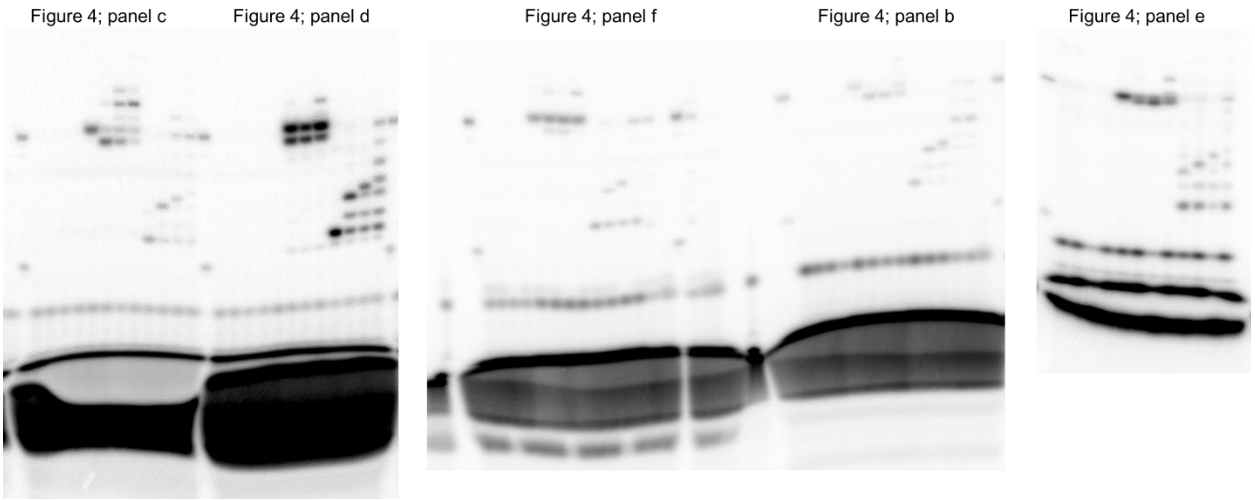

Supplementary Figure 8. Uncropped autoscaled images for panels in Figure 4.

References.

1. **Pettersen, E.F. et al. UCSF Chimera--a visualization system for exploratory research and analysis. *Journal of computational chemistry* 25, 1605-1612 (2004).**
2. **Notredame, C., Higgins, D.G. & Heringa, J. T-Coffee: A novel method for fast and accurate multiple sequence alignment. *Journal of molecular biology* 302, 205-217 (2000).**
3. **Robert, X. & Gouet, P. Deciphering key features in protein structures with the new ENDscript server. *Nucleic acids research* 42, W320-324 (2014).**
4. **Appleby, T.C. et al. Structural basis for RNA replication by the hepatitis C virus polymerase. *Science* 347, 771-775 (2015).**
